# Detection of QTL controlling feed efficiency and excretion in chickens fed a wheat-based diet

**DOI:** 10.1186/s12711-015-0156-y

**Published:** 2015-09-25

**Authors:** Sandrine Mignon-Grasteau, Nicole Rideau, Irène Gabriel, Céline Chantry-Darmon, Marie-Yvonne Boscher, Nadine Sellier, Marie Chabault, Elisabeth Le Bihan-Duval, Agnès Narcy

**Affiliations:** INRA, UR83 Recherches Avicoles, 37380 Nouzilly, France; INRA, LABOGENA, Domaine de Vilvert, 78352 Jouy en Josas Cedex, France; INRA, UE1295 PEAT, 37380 Nouzilly, France

## Abstract

**Background:**

Improving feed efficiency is a major goal in poultry production in order to reduce production costs, increase the possibility of using alternative feedstuffs and decrease the volume of manure. However, in spite of their economic and environmental impact, very few quantitative trait loci (QTL) have been reported on these traits. Thus, we undertook the detection of QTL on 820 meat-type chickens from a F2 cross between D− and D+ lines that were divergently selected on low or high digestive efficiency at 3 weeks of age. Birds were measured for growth between 0 and 23 days, feed intake and feed conversion ratio between 9 and 23 days, breast and abdominal fat yields at 23 days, and the anatomy of their digestive tract (density, relative weight and length of the duodenum, jejunum, ileum, and ratio of proventriculus to gizzard weight) was examined. To evaluate excretion traits, fresh and dry weight, water content, pH, nitrogen to phosphorus ratio from 0 to 23 days, and pH of gizzard and jejunum contents at 23 days were measured. A set of 3379 single nucleotide polymorphisms distributed on 28 *Gallus gallus* (GGA) autosomes, the Z chromosome and one unassigned linkage group was used for QTL detection.

**Results:**

Using the QTLMap software developed for linkage analyses by interval mapping, we detected 16 QTL for feed intake, 13 for feed efficiency, 49 for anatomy-related traits, seven for growth, six for body composition and ten for excretion. Nine of these QTL were genome-wide significant (four for feed intake on GGA1, one for feed efficiency on GGA2, and four for anatomy on GGA1, 2, 3 and 4). GGA16, 19, and 26 carried many QTL for different types of traits that co-localize at the same position.

**Conclusions:**

This study identified several QTL regions that are involved in the control of digestive efficiency in chicken. Further studies are needed to identify the genes that underlie these effects, and to validate these in other commercial populations and for different breeding environments.

**Electronic supplementary material:**

The online version of this article (doi:10.1186/s12711-015-0156-y) contains supplementary material, which is available to authorized users.

## Background

Feed represents the major proportion of production costs for meat-type chickens, i.e. it ranges from 55 to 65 % depending on the production type [1], and costs of poultry diets have increased for several years. This trend is expected to continue with the continuous growth of the world human population that will simultaneously lead to increased needs for poultry meat and for crops that are required for both animal and human consumption. To reduce feed costs and improve feed efficiency, laying hens and meat-type chickens have been selected on traits such as feed conversion ratio (FCR) and residual feed intake (RFI) [2–5]. However, in spite of the very considerable economic impact of these traits, very few quantitative trait loci (QTL) have been reported to date. In fact, among the 4337 QTL that have been detected so far in poultry (laying hens and broilers), only 26 are related to feed efficiency and 11 to feed intake (http://www.animalgenome.org/cgi-bin/QTLdb, 02/10/2014). Moreover, the modern genotypes of broilers have been selected on high-quality feedstuffs that are easily digested by all birds. With these diets, it is not possible to distinguish birds with a high or a low capacity to digest poor quality diets, although this trait will become a very important factor to consider in selection with alternative feedstuffs being increasingly added to poultry diets. Feeding birds with wheat-based diets instead of corn-based diets is a way to challenge their digestive efficiency, since the characteristics of wheat such as hardness and viscosity associated to non-starch polysaccharides content make it much more difficult to digest than corn.

The consequence of poor feed efficiency is increased excretion and hence increased environmental impact of poultry production. It has been shown that the genetic correlations between feed efficiency and relative excreta weight, and nitrogen or phosphorus excretion rate were high (0.66–0.95 [6]) but no report has yet been published on the QTL for these excretion-related traits.

Feed efficiency is a complex trait including feed intake, digestive efficiency and metabolic efficiency to produce new tissues from digested nutrients. In an earlier study, we showed that the digestive component was highly heritable, provided that birds were fed a challenging diet [7, 8]. Using the D+ and D− lines that were divergently selected on high or low digestive efficiency, Tran et al. [9] detected several QTL regions that control digestive efficiency, and showed that, in several cases, these QTL co-localized for digestive efficiency and for anatomy of the digestive tract. However, a large number of traits recorded with this design have not been analyzed yet.

Thus, our aim was to search for QTL for feed efficiency, its components (i.e. feed intake and body composition), and its consequences (i.e. excretion traits). In order to improve the understanding of the relationships between feed efficiency and the anatomy of the digestive tract, we also included a detailed study of the gastro-intestinal tract anatomy.

## Methods

### Animals

All animal care and experimental procedures reported in this paper were in accordance with French and European regulations concerning animal experimentation, including authorization no. 37–100 from the French Ministry of Agriculture. The experimental unit where birds were kept is registered by the ministry of Agriculture with license number C-37-175-1 for animal experimentation. Measures of digestive efficiency in individual cages, blood sampling procedures for genotyping and euthanasia procedures by injection of pentobarbital were approved by the ethics committee in Animal Experimentation of Val de Loire (00886.02 and 01047.02). This ethics committee is registered by the National Committee under the number C2EA-19. The personal license number from the French Veterinary Service for this study is 548.

Chickens from the D+ and D− lines that were divergently selected on high or low digestive efficiency at 3 weeks of age, respectively [8], were crossed at generation 8 of selection to produce an F2 design. The F2 generation consisted of 820 animals that originated from six sires and 60 F1 dams (50 % from the cross between D+ males and D− females, and 50 % from the cross between D− males and D+ females). Five batches of chicks were produced between January and June 2010. The initial population on which the selection experiment was performed is a pure line of broilers, used in a commercial cross that is dedicated to medium-growth broiler production.

From hatching to 10 days of age, birds were reared in one group on the floor, and then they were transferred to individual cages. Throughout the experiment, birds were fed a diet similar to the diet that contains 55 % Rialto wheat and that was used during the selection experiment [9]. This variety of wheat has been chosen for the selection experiment because it is very hard and viscous, and thus especially difficult to digest.

### Growth, feed intake and efficiency

Birds were weighed at hatching and at 9, 14, 17, 20, and 23 days (BWX for body weight at X days). The results for BW23 were published in Tran et al. [9] and will not be presented here. For all F2 birds, feed intake was individually recorded between 9 and 14 days, 14 and 17 days, 17 and 20 days, and 20 and 23 days (FI9_14, FI14_17, FI17_20, and FI20_23). The total feed intake over the experimental period (FI9_23) was calculated as the sum of FI9_14, FI14_17, FI17_20, and FI20_23. Dry matter content of the feed was measured to calculate the dry matter intake during the same periods (FIDM9_14, FIDM14_17, FIDM17_20, FIDM20_23, and FIDM9_23). Residual feed intake was calculated as the difference between measured feed intake and the feed intake estimated by linear regression on metabolic body weight and weight gain during the same time periods (RFI9_14, RFI14_17, RFI17_20, RFI20_23, and RFI9_23). The feed conversion ratio was calculated as the ratio of raw feed intake (FCR9_14, FCR14_17, FCR17_20, FCR20_23, and FCR9_23) or dry matter feed intake (FCRDM9_14, FCRDM14_17, FCRDM17_20, FCRDM20_23, and FCRDM9_23) to weight gain during the same periods. At 23 days, feed was removed for 8 h and birds were fed again for 150 min before slaughter. Their feed intake was recorded during this period and denoted FIS. Elementary statistics for these traits are in Additional file 1: Table S1 and Additional file 2: Table S2.

### Excretion traits

A balance trial was performed between 17 and 20 days to measure excreta weight, digestibility and N and P excretion. Total excreta were weighed and dried to obtain both fresh excreta weight (FEW) and dry excreta weight (DEW). Fresh and dry excreta weights relative to feed intake between 17 and 20 days were calculated (FEW/FI, DEW/FI).

The physico-chemical characteristics of excreta are important parameters that influence the development of gut bacteria in the young, and thus the chemical transformation of excreta into manure. We therefore measured the excreta water content (WC) as the difference between FEW and DEW divided by FEW. Excreta were collected again between 20 and 21 days and homogenized to measure the pH of excreta (PHE) with a portable pH meter equipped with a Xerolyte electrode (model 506, Crison Instruments, SA, Spain).

The nitrogen to phosphorus excretion ratio (N/P) was calculated as an indicator of the equilibrium of the composition of excreta as manure. N excretion was measured for all birds using near-infrared spectrophotometry (NIRS; Foss NIRSystems, Inc., Silver Spring, MD, USA), using the method of Bastianelli et al. [10]. P excretion was measured by colorimetric analysis. Elementary statistics for these traits are in Additional file 1: Table S1.

### Body composition, anatomy of digestive tract and pH of gizzard and intestinal contents

Birds were slaughtered at 23 days and the gizzard, proventriculus, duodenum (pancreatic loop), jejunum (from the pancreatic ducts to Meckel’s diverticulum) and ileum (from Meckel’s diverticulum to ileocecal junction) were removed, emptied and weighed (GW, PRW, DUW, JEW, ILW, respectively, in g). The total weight of the small intestine (INW) was calculated as the sum of DUW, JEW, and ILW. For all segments of the digestive tract, organ weights (OW) were also expressed as values relative to body weight (OW/BW) or as values relative to feed intake (OW/FI). Similarly, we measured the length of the duodenum, jejunum, ileum and total intestine on a raw basis (DUL, JEL, ILL, INL, in cm) and on a basis relative to body weight (DUL/BW, JEL/BW, ILL/BW, INL/BW, in cm g^−1^) and to feed intake (DUL/FI, JEL/FI, ILL/FI, INL/FI, in cm g^−1^). Intestine density was calculated as the ratio of intestine weight to intestine length for the duodenum, jejunum, ileum and total intestine (DUD, JED, ILD, IND, in g cm^−1^). Since proventricular dilatation was observed in this population, the ratio of proventriculus to gizzard weight was also calculated (PRW/GW, g g^−1^). Gizzard, proventriculus and total intestine weights (raw data and values relative to body weight) as well as intestine length relative to body weight and intestine density were presented in Tran et al. [9] and are not discussed further in this paper.

Breast meat (*Pectoralis major* and *Pectoralis minor*) and abdominal fat were also removed and their weights relative to the body weight at 23 days were measured (BRY, AFY, in g g^−1^).

Finally, since the digesta pH in the different compartments of the digestive tract influences activity and secretion of digestive enzymes, we also measured the pH in gizzard and jejunum contents, by collecting boluses at slaughter. The pH was then measured with a portable pH meter equipped with a Xerolyte electrode (model 506, Crison Instruments, SA, Spain) (PHG, PHJ).

Elementary statistics for these traits are shown in Additional file 3: Tables S3 and Additional file 4: Table S4.

### Markers and genotyping

All F0, F1 and F2 birds were genotyped with a dedicated Illumina Infinium custom array including 6000 single nucleotide polymorphisms (SNPs) that were selected for their informativity in our design and distribution across the genome [9]. SNPs that deviated from the Hardy–Weinberg equilibrium within families or that showed inconsistent genotyping data relative to pedigree or genetic map information and poor quality SNPs were discarded from the analysis in order to reduce the risk of erroneous results [9]. Finally, 3379 SNPs were used. The genetic map was deduced from the physical position of the SNPs and from the genetic consensus reference map published by Groenen et al. [11]. This set of SNPs covers 3099.1 cM.

### QTL analysis

QTL detection was carried out with the QTLMap software [12] using a half-sib model [13, 14] with interval mapping based on maximum likelihood estimations [15]. All data were analyzed in a single model but QTL effects were estimated for each sire family. This model does not make assumptions on the number of QTL alleles segregating in the design. The traits were analyzed separately. Depending on a preliminary analysis of the variance, data were pre-corrected for fixed effects of batch (four levels), sex (two levels), rearing cell (three levels), cage row (three levels), slaughter time (morning or afternoon), experimenter at slaughter (i.e. the person in charge of cutting intestinal segments, seven levels). QTL analyses were performed by comparing the hypothesis of one QTL (H1) versus no QTL (H0) to test the segregation of a QTL on each linkage group. For chromosome Z, separate analyses were performed for males and females, and are presented as Zm and Zf, respectively.

For each trait on each chromosome, the significance threshold at the chromosome-wide level was calculated from the results of 5000 simulations of performance under the null hypothesis. For the most significant QTL, 20,000 simulations were performed to derive the genome-wide *p* value (P_G_) from the chromosome-wide *p* value (P_C_) using an approximate Bonferroni correction:

$${\text{P}}_{\text{G}} = 1 - (1 - {\text{P}}_{\text{C}} )^{{{ 1\mathord{\left/ {\vphantom { 1{\text{r}}}} \right. \kern-0pt} {\text{r}}}}} ,$$

where r is the ratio of the length of a specific chromosome to the length of the genome considered for QTL detection as in Tilquin et al. [16]. Confidence intervals for QTL (95 %) were estimated using the LOD drop-off method as proposed by Lander and Botstein [15].

The significance of the QTL effects within each sire family was tested using a Student test, by assuming an equal distribution of the QTL alleles in the progeny. A QTL effect was retained as significant for Student test *p* values less than 0.05, and the corresponding sire families were assumed to segregate for this QTL. These familial substitution effects were estimated in families found to segregate significantly for the QTL.

## Results

### Number and positions of the QTL

**Fig. 1:**
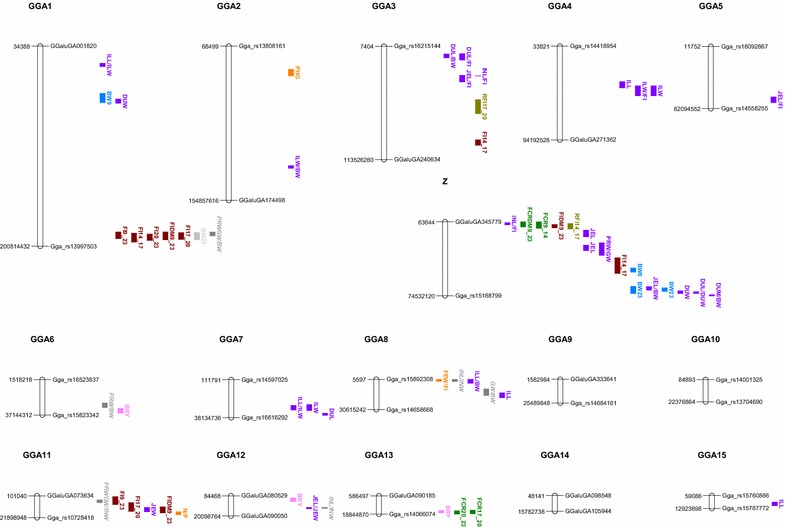
Map of QTL^1,2^ on chromosomes 1–15 and chromosome Z. ^1^Results from Tran et al. [9]; FIX_Y, FIDMX_Y: raw and dry matter feed intake between X and Y days; FCRX_Y, FCRDMX_Y: raw and dry matter feed conversion ratio between X and Y days; FEW, DEW: fresh and dry excreta weight between 17 and 20 days; FEW/FI, DEW/FI: fresh and dry excreta weight relative to feed intake between 17 and 20 days; WC: water content of excreta, N/P: nitrogen to phosphorus ratio in excreta; DUW, JEW, ILW, INW^1^: duodenum, jejunum, ileum, and small intestine weight at 23 days; DUW/BW, JEW/BW, ILW/BW, INW/BW^1^: duodenum, jejunum, ileum, and small intestine weight relative to body weight at 23 days; DUW/FI, JEW/FI, ILW/FI, INW/FI: duodenum, jejunum, ileum, and small intestine weight at 23 days relative to feed intake between 9 and 23 days; DUL, JEL, ILL, INL^1^: duodenum, jejunum, ileum, and small intestine length at 23 days; DUL/BW, JEL/BW, ILL/BW, INL/BW^1^: duodenum, jejunum, ileum, and small intestine length relative to body weight at 23 days; DUL/FI, JEL/FI, ILL/FI, INL/FI: duodenum, jejunum, ileum, and small intestine length at 23 days relative to feed intake between 9 and 23 days; DUD, JED, ILD, IND^1^: duodenum, jejunum, ileum, and small intestine density at 23 days; PHE, PHG, PHJ: pH of excreta, gizzard and jejunum contents; BWX^1^: body weight at X days; AMEn^1^: metabolizable energy corrected to zero nitrogen retention; CDUDM^1^, CDUP^1^, CDUS^1^: coefficients of digestive use of dry matter, protein and starch; PRW/BW^1^, GW/BW^1^, PRWGW/BW^1^: proventriculus, gizzard and proventriculus plus gizzard weights relative to body weight. ^2^Feed intake (*dark red*), feed efficiency (*dark green*), residual feed intake (*olive green*), excretion traits (*orange*), digestive efficiency (*black*), body weight (*blue*, *light grey*), breast and fat yields (*pink*) and anatomy of the digestive tract (*purple*, *dark grey*)

**Fig. 2:**
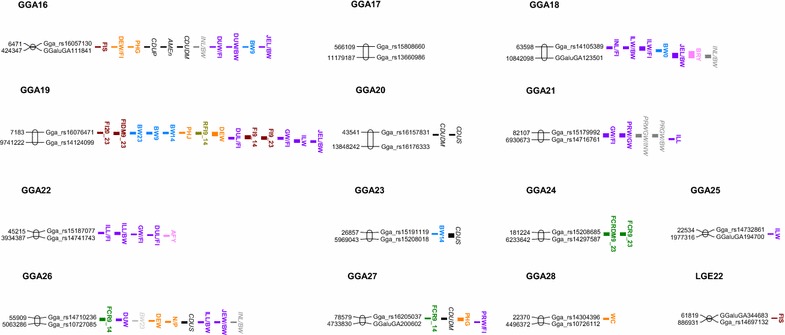
Map of QTL^1,2^ on chromosomes 16 to 28 and linkage group LGE22C19. ^1^Results from Tran et al. [9]; FIX_Y, FIDMX_Y: raw and dry matter feed intake between X and Y days; FCRX_Y, FCRDMX_Y: raw and dry matter feed conversion ratio between X and Y days; FEW, DEW: fresh and dry excreta weight between 17 and 20 days; FEW/FI, DEW/FI: fresh and dry excreta weight relative to feed intake between 17 and 20 days; WC: water content of excreta, N/P: nitrogen to phosphorus ratio in excreta; DUW, JEW, ILW, INW^1^: duodenum, jejunum, ileum, and small intestine weight at 23 days; DUW/BW, JEW/BW, ILW/BW, INW/BW^1^: duodenum, jejunum, ileum, and small intestine weight relative to body weight at 23 days; DUW/FI, JEW/FI, ILW/FI, INW/FI: duodenum, jejunum, ileum, and small intestine weight at 23 days relative to feed intake between 9 and 23 days; DUL, JEL, ILL, INL^1^: duodenum, jejunum, ileum, and small intestine length at 23 days; DUL/BW, JEL/BW, ILL/BW, INL/BW^1^: duodenum, jejunum, ileum, and small intestine length relative to body weight at 23 days; DUL/FI, JEL/FI, ILL/FI, INL/FI: duodenum, jejunum, ileum, and small intestine length at 23 days relative to feed intake between 9 and 23 days; DUD, JED, ILD, IND^1^: duodenum, jejunum, ileum, and small intestine density at 23 days; PHE, PHG, PHJ: pH of excreta, gizzard and jejunum contents; BWX: body weight at X days; AMEn^1^: metabolizable energy corrected to zero nitrogen retention; CDUDM^1^, CDUP^1^, CDUS^1^: coefficients of digestive use of dry matter, protein and starch; PRW/BW^1^, GW/BW^1^, PRWGW/BW^1^: proventriculus, gizzard and proventriculus plus gizzard weights relative to body weight ^2^Feed intake (*dark red*), feed efficiency (*dark green*), residual feed intake (*olive green*), excretion traits (*orange*), digestive efficiency (*black*), body weight (*blue*, *light grey*), breast and fat yields (*pink*) and anatomy of the digestive tract (*purple*, *dark grey*)

We detected 16 QTL for feed intake, five for residual feed intake, and eight for feed conversion ratio (Table 1; Figs. 1, 2). Five of these QTL (on GGA1 and Z) were genome-wide significant and three other QTL (on GGA13 and Z) were close to genome-wide significance (P_G_ between 0.11 and 0.13). We also detected ten QTL for excretion traits (Table 2; Figs. 1, 2), none of them being genome-wide significant: four for pH in the gizzard or in the jejunum, three for the quantity of excreta, two for the ratio of nitrogen to phosphorus in excreta, and one for water content of excreta. On GGA2, the QTL for pH in the gizzard was close to genome-wide significance (P_G_ = 0.15).

**Table 1:** QTL detected for feed efficiency and feed consumption

Chr	Trait^a^	Position (cM)	CI^b^	Flanking markers of CI		Level of significance		QTL effect^d^ (NF^e^)
						Chr-wide	Genome-wide^c^	
1	FI9_23	457.0	453.0–469.0	Gga_rs13986943	Gga_rs15546764	0.003	0.050	0.383 (2)F^g^
	FI14_17	460.0	456.1–476.5	Gga_rs13988066	Gga_rs14936549	0.050	NS	0.359 (2)F
	FI20_23	464.0	458.0–473.0	GgaluGA060547	Gga_rs10729419	0.001	0.010	0.359 (2)
	FIDM9_23	464.0	453.0–472.0	Gga_rs13986943	Gga_rs15548184	0.001	0.010	0.276 (4)
	FI17_20	467.0	455.0–470.0	Gga_rs13987585	GGaluGA062319	0.001	0.010	0.252 (6)
3	RFI17_20	141.0	127.0–159.0	Gga_rs16276740	Gga_rs14369065	0.044	NS	0.242 (4)
	FI14_17	230.0	222.0–234.0	GGaluGA233748	GGaluGA235233	0.029	NS	0.210 (5)F
10	RFI14_17	89.0	85.0–90.0	Gga_rs10723404	Gga_rs13704690	0.035	NS	0.226 (4)F
11	FI9_23	18.0	3.0–28.0	GGaluGA074015	Gga_rs14022155	0.035	NS	0.175 (5)
	FI17_20	27.0	22.0–49.0	Gga_rs14020873	Gga_rs15620835	0.029	NS	0.240 (3)
	FIDM9_23	49.0	35.0–54.0	Gga_rs14697690	Gga_rs16740226	0.037	NS	0.191 (4)
13	FCR20_23	52.5	50.0–60.0	GGaluGA097117	Gga_rs15712088	0.030	NS	0.256 (3)
	FCR17_20	53.5	48.0–59.0	Gga_rs1999041	Gga_rs14065910	0.003	0.130	0.303 (3)
16	FIS	0.0^f^	–	Gga_rs16057130	Gga_rs15788030	0.032	NS	0.210 (4)
19	FI20_23	0.0^f^	0.0–6.3	Gga_rs16076471	Gga_rs14116110	0.005	NS	0.202 (6)F
	FIDM9_23	0.0^f.^	0.0–12.5	Gga_rs16076471	GGaluGA125050	0.033	NS	0.243 (3)F
	RFI9_14	5.0	0.0–10.1	Gga_rs16076471	Gga_rs15838137	0.050	NS	0.181 (5)F
	FI9_14	27.0	17.9–33.3	Gga_rs14118744	Gga_rs14121640	0.015	NS	0.227 (4)
	FI9_23	29.0	21.8–36.4	GGaluGA126160	GGaluGA127728	0.003	NS	0.190 (6)
24	FCRDM9_23	9.0	0.0–20.7	Gga_rs15208685	Gga_rs15217571	0.003	NS	0.264 (4)
	FCR9_23	9.0	0.0–20.5	Gga_rs15208685	Gga_rs15217571	0.003	NS	0.338 (3)
26	FCR9_14	6.0	0.0–21.6	Gga_rs14710236	Gga_rs13606082	0.013	NS	0.388 (2)F
27	FCR9_14	0.0^f^	0.0–11.7	Gga_rs16205037	Gga_r16207282	0.049	NS	0.195 (4)
LGE22	FIS	2.0	0.0–2.0	GGaluGA344683	Gga_rs13742310	0.040	NS	0.331 (2)
Zf	FCRDM9_23	8.5	0.0–14.8	Gga_rs15994240	Gga_rs15249807	0.008	0.110	2.197 (2)F
Zm	FCR9_14	14.5	0.0–19.2	Gga_rs15994240	Gga_rs14689442	0.049	NS	0.877 (3)
Zf	FIDM9_23	16.5	8.6–18.8	GGaluGA346525	Gga_rs14689342	0.009	0.120	1.192 (4)
Zf	RFI14_17	21.5	6.5–22.6	Gga_rs14067631	Gga_rs14783328	0.001	0.020	0.967 (4)
Zf	FI14_17	140.5	113.8–160.5	Gga_rs16110590	Gga_rs14769351	0.014	NS	0.774 (5)

^a^FIX_Y: feed intake between X and Y days; FIDMX_Y: dry matter feed intake between X and Y days; FCRX_Y: feed conversion ratio between X and Y days; FCRDMX_Y: feed conversion ratio between X and Y days on a dry matter basis

^b^1-LOD-drop off confidence interval (lower and upper boundaries, cM)

^c^NS: P > 0.150 at the genome wide level

^d^QTL effect as a proportion of the phenotypic standard deviation of trait

^e^Number of F1 sire families heterozygous for the QTL (P < 0.05, Student test)

^f^QTL located at the telomere

^g^F: QTL fixed in F1 sire families in which it is significant

**Table 2:** QTL detected for excretion-related traits

Chr	Trait^a^	Position (cM)	CI^b^	Flanking markers of CI		Level of significance		QTL effect^d^ (NF^e^)
						Chr-wide	Genome-wide^c^	
2	PHG	48.0	47.4–60.2	Gga_rs14152021	Gga_rs14158104	0.025	0.150	0.241 (4)F^g^
8	FEW/FI	4.0	0–5.3	Gga_rs158923080	Gga_rs16617921	0.027	NS	0.178 (5)
11	N/P	55.0	51.0–60.0	Gga_rs14029626	Gga_rs15623939	0.047	NS	0.177 (4)
16	PHG	2.0	–	Gga_rs16057130	Gga_rs15788030	0.047	NS	0.186 (4)
19	PHJ	0.0^f^	0.0–5.5	Gga_rs16076471	GGaluGA124279	0.048	NS	0.254 (2)
	DEW	9.0	0.0–18.5	Gga_rs16076471	Gga_rs15842421	0.050	NS	0.161 (3)
26	DEW	25.0	20.7–27.5	Gga_rs16201345	GGaluGA196847	0.042	NS	0.248 (2)
	N/P	34.0	28.1–37.0	GGaluGA196847	Gga_rs14300646	0.010	NS	0.184 (5)
27	PHG	19.7	0.0–32.1	Gga_rs16205037	GGaluGA199670	0.045	NS	0.214 (3)
28	WC	8.0	0.0–14.1	Gga_rs14304396	Gga_rs16211067	0.030	NS	0.171 (5)

^a^FEW, DEW: fresh and dry excreta weight between 17 and 20 days; FEW/FI, DEW/FI: fresh and dry excreta weight between 17 and 20 days relative to feed intake between 17 and 20 days; PHE: pH in the excreta; WC: water content of excreta, N/P: nitrogen to phosphorus ratio in excreta

^b^1-LOD-drop off confidence interval (lower and upper boundaries, cM)

^c^NS: P > 0.150 at the genome wide level

^d^QTL effect as a proportion of the phenotypic standard deviation of trait

^e^Number of F1 sire families heterozygous for the QTL (P < 0.05, Student test)

^f^QTL located at the telomere

^g^F:QTL fixed in F1 sire families in which it is significant

We found 49 QTL for different anatomy traits (Table 3; Figs. 1, 2). Among these 49 QTL, four were genome-wide significant for duodenum and ileum weight or length (on GGA1, 2, 3, and 4). Eight other QTL for the weight and length of intestinal segments and for the ratio of proventriculus to gizzard weight were close to significance on GGA4 and GGAZ (P_G_ between 0.06 and 0.13). Finally, we also detected seven QTL for body weight and five for body composition, among which the QTL for breast yield on GGA6 was close to genome-wide significance (P_G_ = 0.07).

**Table 3:** QTL detected for anatomy traits

Chr	Trait^a^	Position (cM)	CI^b^	Flanking markers of CI		Level of significance		QTL effect^d^ (NF^e^)
						Chr-wide	Genome-wide^c^	
1	ILD	46.0	41.8–49.9	Gga_rs13828473	Gga_rs0072444	0.034	NS	0.262 (5)
	BW9	133.0	116.0–137.0	Gga_rs14818416	GgaluGA019034	0.017	NS	0.313(3)
	DUW	134.0	129.5–138.5	Gga_rs13865892	Gga_rs13654276	0.004	0.020	0.235 (4)F^g^
2	ILW/BW	246.0	243.3–247.3	Gga_rs14239031	Gga_rs14240625	0.004	0.030	0.245 (5)
3	DUL/BW	24.0	19.8–26.9	Gga_rs14316939	Gga_rs15262904	<0.0001	0.010	0.242 (5)
	DUL/FI	24.0	17.9–32.1	GgaluGA205532	Gga_rs15755832	0.043	NS	0.330 (2)F
	JEL/FI	72.0	69.5–84.7	Gga_rs13721073	GgaluGA216152	0.043	NS	0.247 (4)
	INL/FI	72.0	70.2–74.4	Gga_rs13721291	Gga_rs14332443	0.031	NS	0.267 (4)
4	ILL	82.0	77.0–88.0	GgaluGA253242	GgaluGA255382	0.002	0.020	0.211 (5)
	ILW/FI	98.0	87.0–105.0	GgaluGA254623	GgaluGA257921	0.090	0.100	0.270 (4)
	ILW	98.0	87.0–103.0	GgaluGA254623	GgaluGA257921	0.011	0.110	0.308 (3)F
5	JEW/FI	133.0	129.0–143.0	Gga_rs14544598	Gga_rs14551270	0.034	NS	0.269 (3)F
6	BRY	97.0	91.0–103.0	Gga_rs14590462	Gga_rs 15817647	0.003	0.070	0.209 (4)
7	ILD	85.0	77.0–89.0	GgaluGA316720	GgaluGA318716	0.041	NS	0.177 (5)
	ILW	85.0	74.0–91.0	GgaluGA316334	GgaluGA318885	0.043	NS	0.199 (4)
	DUL	102.0	99.0–105.0	Gga_rs15883120	GgaluGA320562	0.040	NS	0.216 (5)
8	ILW/BW	4.0	0.0–11.0	Gga_rs15892308	Gga_rs15899734	0.029	NS	0.244 (3)
	ILL	52.0	41.0–55.0	Gga_rs15911337	Gga_rs15920212	0.022	NS	0.197 (4)
11	JEW	41.0	37.0–50.0	Gga_rs14024860	GgaluGA078344	0.032	NS	0.177 (5)
12	BRY	18.0	8.0–21.0	Gga_rs15632811	Gga_rs14974160	0.035	NS	0.193 (5)
	JED	44.0	43.0–46.0	Gga_rs15654987	Gga_rs14042499	0.010	NS	0.256 (3)
13	BRY	50.5	49.0–55.0	Gga_rs14063011	Gga_rs14064923	0.043	NS	0.175 (4)
15	ILL	32.4	28.4–43.4	GgaluGA108494	Gga_rs14094799	0.010	NS	0.212 (3)
16	DUW/FI	0.0^f^	–	Gga_rs16057130	Gga_rs15788030	0.035	NS	0.214 (3)
	DUW/BW	1.0	–	Gga_rs16057130	Gga_rs15788030	0.001	NS	0.200 (5)
	JEL/BW	2.0	–	Gga_rs16057130	Gga_rs15788030	0.044	NS	0.261 (2)
16	BW9	2.0	–	Gga_rs16057130	Gga_rs15788030	0.043	NS	0.204 (2)
18	ILW/FI	5.3	0.0–16.3	Gga_rs14105389	Gga_rs14107424	0.009	NS	0.212 (4)
	ILW/BW	5.3	0.7–9.4	Gga_rs14105389	Gga_rs14107424	0.029	NS	0.219 (3)
	BW0	14.3	12.9–22.4	GgaluGA118988	Gga_rs13507599	0.044	NS	0.173 (4)
	JEL/BW	36.3	30.3–51.3	Gga_rs15825400	Gga_rs16348086	0.022	NS	0.240 (4)
	BRY	37.3	22.0–51.0	Gga_rs14110277	Gga_rs16348086	0.050	NS	0.208 (4)F
19	BW9	5.0	0.0–9.8	Gga_rs16076471	GgaluGA124727	0.021	NS	0.177 (5)
	BW14	5.0	0.0–7.8	Gga_rs16076471	Gga_rs14116374	0.020	NS	0.189 (5)
	DUL/FI	27.0	22.0–35.0	GgaluGA126497	Gga_rs15049206	0.028	NS	0.189 (5)
	GW/FI	31.0	25.0–36.0	GgaluGA126497	GgaluGA127728	0.032	NS	0.171 (6)
	ILW	45.0	38.2–52.0	Gga_rs15850796	Gga_rs14124099	0.029	NS	0.204 (4)
	JEL/BW	45.0	44.3–52.0	GgaluGA128413	Gga_rs14124099	0.030	NS	0.269 (2)
21	GW/FI	24.0	7.8–34.1	GgaluGA182305	Gga_rs14284716	0.018	NS	0.244 (3)
	ILL	51.0	44.8–52.0	Gga_rs10726959	Gga_rs14716761	0.045	NS	0.219 (3)
22	ILL/FI	4.0	0.0–22.7	Gga_rs14187077	Gga_rs14690641	0.018	NS	0.272 (3)
	ILL/BW	21.0	0.0–34.3	Gga_rs14187077	Gga_rs16688369	0.027	NS	0.193 (4)
	GW/FI	33.0	25.0–37.0	Gga_rs16688631	GgaluGA186231	0.046	NS	0.264 (2)
	DUL/FI	44.0	38.0–50.0	GgaluGA186231	GgaluGA186550	0.032	NS	0.290 (3)
	AFY	58.0	47.0–61.0	GgaluGA186448	Gga_rs14741743	0.045	NS	0.244 (2)
23	BW14	11.0	6.8–12.5	GgaluGA187410	Gga_rs14289024	0.050	NS	0.205 (4)
25	ILW	54.0	45.0–62.0	GgaluGA194347	Gga_rs16048502	0.040	NS	0.181 (4)
26	DUW	20.0	6.4–23.4	Gga_rs15467187	Gga_rs13606162	0.003	NS	0.216 (3)
	ILL/BW	37.0	30.3–40.0	Gga_rs13606421	Gga_rs16204548	0.005	NS	0.222 (2)
	JEL/BW	37.0	34.0–40.0	Gga_rs15235010	Gga_rs14300958	0.050	NS	0.270 (2)
27	PRW/FI	50.0	45.5–54.0	Gga_rs15243050	GgaluGA200602	0.035	NS	0.200 (4)
Zf	INL/FI	7.0	4.0–9.0	Gga_rs15993613	GgaluGA346550	0.034	NS	0.382 (3)F
Zm	JEL	33.0	26.0–47.0	Gga_rs15243050	Gga_rs14757928	0.010	0.130	0.331 (4)
Zf	JEL	80.0	73.0–89.0	Gga_rs13734017	Gga_rs14760912	0.004	0.060	0.323 (5)
Zf	PRW/GW	82.0	65.0–104.0	GgaluGA349288	Gga_rs16106986	0.008	0.100	0.348 (3)
Zf	JEL/BW	207.0	203.0–213.0	Gga_rs14776035	Gga_rs16125407	0.010	0.130	0.284 (4)
Zm	BW0	210.5	207.0–217.0	Gga_rs29004800	Gga_rs16126655	0.042	NS	0.844 (3)
Zf	DUW	221.0	216.0–224.0	Gga_rs16126120	Gga_rs15992576	0.006	0.080	0.343 (5)
Zf	DUL/DUW	222.0	219.0–224.0	Gga_rs14781920	Gga_rs15992576	0.004	0.060	0.317 (5)
Zm	DUL/BW	230.0	228.4–231.0	Gga_rs15990597	Gga_rs15168799	0.009	0.120	0.304 (4)

^a^DUW, JEW, ILW, INW: duodenum, jejunum, ileum, and small intestine weight at 23 days; DUW/BW, JEW/BW, ILW/BW, INW/BW: duodenum, jejunum, ileum, and small intestine weight at 23 days relative to body weight at 23 days; DUW/FI, JEW/FI, ILW/FI, INW/FI: duodenum, jejunum, ileum, and small intestine weight at 23 days relative to feed intake between 9 and 23 days; DUL, JEL, ILL, INL: duodenum, jejunum, ileum, and small intestine length at 23 days; DUL/BL, JEL/BL, ILL/BL, INL/BL: duodenum, jejunum, ileum, and small intestine length at 23 days relative to body weight at 23 days; DUL/FI, JEL/FI, ILL/FI, INL/FI: duodenum, jejunum, ileum, and small intestine length at 23 days relative to feed intake between 9 and 23 days; DUD, JED, ILD, IND: duodenum, jejunum, ileum, and small intestine density at 23 days; PHG, PHJ: pH in the content of gizzard and jejunum; BWX: body weight at X days

^b^1-LOD-drop off confidence interval (lower and upper boundaries, in cM)

^c^NS: P > 0.150 at the genome wide level

^d^QTL effect as a proportion of the phenotypic standard deviation of trait

^e^Number of F1 sire families heterozygous for the QTL (P < 0.05, Student test)

^f^QTL located at the telomere

^g^F: QTL fixed in F1 sire families in which it is significant

### Co-localizations between QTL

We observed two types of co-localization between QTL (Figs. 1, 2). On the one hand, on GGA1, 13, 19, 24, and Z, we found QTL at the same position for traits that are very similar, but measured at different ages or on a different basis (i.e., on a raw or dry matter basis). For example, this was the case on GGA1 for five QTL for feed intake, measured between 9 and 23 days (on raw or dry matter basis), between 14 and 17 days, 17 and 20 days, and 20 and 23 days. Similarly, for the anatomy of the digestive tract, four to five QTL co-localized on GGA16, 18, 19, 21, 22, 26 and Z (Fig. 1). Co-localization was also present for body weights at two different ages on GGA19. However, the results obtained for the co-localization on GGA16 have to be regarded with caution, since only two SNPs separated by quite a short distance were detected, which indicates that the precision of localization on this chromosome is poor.

On the other hand, we also observed co-localizations between QTL for different categories of traits. On GGA16, we observed the co-localization of 11 QTL for feed intake, weight and length of intestinal segments, growth and digestion. However, as above, due to the small number of SNPs on this chromosome, these co-localizations have to be regarded with caution. GGA19 was a hot spot in our design, with seven QTL between 0 and 9 cM for feed intake, growth, feed efficiency, digestion, and excretion. Similarly, on GGA26 between 34 and 37 cM, we detected QTL for relative lengths of intestinal segments and excretion at the same position as a QTL for the coefficient of digestive use of starch reported by Tran et al. [9] using the same design. On GGA26, two QTL were also identified between 20 and 25 cM for duodenum weight and excreta weight.

Most of the other regions that presented co-localization of QTL for traits of two different categories included anatomy traits and (1) efficiency (between 3 and 4 cM on GGA8 and between 7.0 and 14.5 cM on GGAZ), (2) feed intake (between 17 and 18 cM on GGA11, between 41 and 49 cM on GGA11, and between 27 and 31 cM on GGA19), (3) growth (between 207.0 and 217.0 cM on GGAZ), and (4) body composition (between 36.3 and 45.0 cM on GGA18).

### Size of QTL effects

All detected QTL were of moderate effect except for the QTL for body weight at 0 day, feed intake and feed efficiency on chromosome Z, with effects that ranged from 0.774 to 2.197 standard deviations. This is partly due to the fact that some QTL were often not fixed in the parental populations (16 out of 101 QTL), and that some QTL were not present in all sire families (four QTL out of 99 were present in the six F1 sire families). The mean effects of fixed and non-fixed QTL were equal to 0.39 and 0.28 standard deviations, respectively.

For most fixed QTL, the origin of the positive allele was consistent with the differences between D+ and D− lines. Birds in the D− line have heavier intestines, consume more food and are less efficient. Indeed, the positive allele of QTL for DUW (GGA1), FI14_17 (GGA1), FI20_23, FIDM9_23, RFI9_14 (GGA19) and FCRDM9_23 (GGAZ) originated from the D− line, for which these traits had higher values. In contrast, alleles that are responsible for higher pH in the gizzard (GGA2), greater residual feed intake (GGA10) and smaller breast yield (GGA18) originated from the D+ line, which is in the opposite direction of the phenotypic differences between these lines.

## Discussion

### Positions of QTL

Among the QTL detected in our study, several co-localized with other QTL reported in the literature. Most of these co-localizations were observed for feed intake in our study, and body weight or growth rate on GGA1, 2, 3 and 11 [17–21], feed efficiency on GGA11 [19] and body composition including fatness on GGA1 and 11 [22, 23], thigh muscle on GGA3 and 11 [18] or proventriculus and gizzard weight on GGA1 [9, 24]. On GGA1, 4 and 13, several co-localizations between our QTL for raw or relative intestine weight with QTL for fatness [22, 25–28] and growth [20, 25, 29] were detected. Since the ileum and jejunum are the key intestine segments for lipid digestibility [30], the co-localization between QTL for fatness and intestine weight and length is not surprising.

GGA16 was a special case since it included 12 QTL for digestive efficiency, anatomy of the digestive tract, excretion traits, body weight and feed intake by combining the results of this study and those of Tran et al. [9] using the same design. Ewald et al. [31] also published a QTL for feed conversion ratio on GGA16. It is difficult to reach a conclusion about the real co-localization of all these QTL since the number of SNPs available on GGA16 is very small.

### Candidate genes

Based on the fact that the D+ and D− lines have been selected on their digestive efficiency, it is not surprising to identify several genes involved in digestive physiology within the QTL regions detected in this study. For example, several genes involved in the secretion of gastric or bile acids that play a role in pH determination in the gizzard and intestine were identified, such as *gastrin*-*releasing peptide* (*GRP*, [32]), *phospholipase A2* (*PLA2*, [33]), *tachykinin 1 and substance P* [34–36], *neurokinin A* and *TAC3* (coding for tachykinin NK3 receptor, [37]). Among these genes, *TAC1* (encoding substance P) is located within a QTL for pH in the gizzard. The QTL regions detected in this study include genes that are related to the different functions of the gastro-intestinal tract. Several genes are involved in muscle contraction, which is consistent with the large difference in transit time observed between D+ and D− lines [38]. In addition to their acid secretion function, gastrin-releasing peptide and tackykinin 1 have an important role in the contraction of the gastrointestinal tract [39]. Detecting such genes is not unexpected since filling and emptying the gastrointestinal tract through its contraction directly affect neuronal stimulation of gastric acid secretion.

We also identified several positional candidate genes that encode enzymes involved in the degradation of lipids (pancreatic lipase PNLIP) or of malic acid (malic enzyme). The latter is located within a QTL for intestine length, which is consistent with a previous report by Ocak et al. [40] who showed that quails supplemented with malic acid have longer intestines. Major differences in size and structure of the gastro-intestinal tract have been previously reported between D+ and D− lines [41, 42]. We found many genes in the QTL regions that are involved in the growth, structure and function of gastro-intestinal tissue. For example, within the QTL for ileum length on GGA4, we found the gene *caspase 3*. This gene contributes to the apoptosis of apical cells of the intestinal epithelium, which in turn stimulates epithelial cell renewal and influences the height of intestinal villi [43]. Another example within the QTL for feed efficiency on GGA13, is the *SLC22A4* gene (*solute carrier family member 4*), which is involved in the metabolism of ergothionein, a protective molecule of the intestine. Knock-out mice for this gene show considerable degradation of the structure of the intestinal villi [44]. Several solute carriers (e.g., *SLC1A1*, *SLC1A4*) involved in the glutamine-glutamate metabolism pathway were identified within some of the QTL of our study, which is consistent with the fact that glutamine is preferred to glucose as a metabolite to provide energy to enterocytes, since intestinal tissue has a high turnover rate and requires a high level energy source [45].

Apart from the intestine cells, the extra-cellular matrix is an essential component of the structure and functionality of the intestine. This matrix includes several components such as elastin, which ensures flexibility and elasticity of the intestine, and collagen and cell junctions to ensure adhesion between cells and strength of the tissue. In this study, we found genes involved in the claudin pathway (*CLDN3*, *CLDN4* and *EPCAM*), which are essential members of the tight junctions between intestinal cells, within the QTL for duodenum length (GGA3) and for residual feed intake, feed intake, growth, pH of jejunum and excreta weight (GGA19) [46, 47]. The same region of GGA19 also contains the *elastin* gene. Finally, several genes linked to the synthesis of collagen were also present, such as *neuroplastin* (within the QTL for ileum length on GGA4, [48]).

More generally, we found many genes within the confidence intervals of the QTL that are involved in the growth of intestinal tissues, cell proliferation and differentiation. Several of these genes are linked to the growth of fibroblasts (*FGF1*, *FGF10*, *SREBF2* or *FGFR2*), support intestine cells [49] and are involved in the synthesis of collagen. Some of these genes, such as *FGF1*, are also involved in angiogenesis, which is essential to the normal function and development of intestinal tissue [50]. Among the genes that have a role in cell proliferation, *glucosylceramide synthase* (*UGCG*) is specifically known to affect enterocyte proliferation [51]. Several genes of the p70S6K and mTor pathway are also present within QTL regions for intestine weight and length and breast meat yield, which is consistent with the role of this pathway in protein synthesis [52].

The intestine has an important function in the protection of the body since it is a barrier against the external environment and thus prevents entry of luminal pathogens and pro-inflammatory molecules in the body. This is consistent with the large number of QTL found on GGA16 that carries the major histocompatibility complex [53]. The role of the intestine in the immune system depends on the secretion of protective mucus on the surface of the intestine, through efficient tight junctions (already mentioned above) and anti-inflammatory responses. *Phospholipase A2* (*PLA2*) within the QTL for duodenum weight and body weight on GGA1 has a protective function through its regulatory role on epithelial secretion of mucus [54]. Several genes involved in inflammatory or anti-inflammatory responses such as interleukines (*IL10*, *IL7R*), solute carriers (*SLC1A1*, *SLC26A9*, *SLC1A14*) and nerve growth factor [55–57] are also present within QTL for intestine length or weight detected in this study.

The last category of identified genes is involved in the regulation of feed intake. The QTL for feed intake on GGA1 includes the *G*-*protein coupled receptor 83* (*GPR72*) gene, which encodes a neuropeptide Y receptor that directly influences feed intake [58, 59], and also the *prolylcarboxypeptidase* (*PCRP*) gene, which decreases secretion of α-melanocyte-stimulating hormone (α-MSH) and thereafter increases feed intake [60]. In addition, *CRHR1* on GGA27 (*corticotrophin releasing hormone receptor 1*) was identified and since it is involved in the regulation of appetite [61], it could impact the pH in the gizzard by stimulating the mechano-sensors involved in the secretion of gastric acids. However, the regulation of energy metabolism through the TGF-β pathway could also be affected since QTL for feed intake and efficiency on GGAZ include the *Fussell18* gene and several genes encoding Smad proteins [62].

### Implications for selection

Although most of the QTL detected in this study have moderate effects, their use in selection may lead to a significant reduction in production costs. For instance, the substitution effect of the QTL that controls feed intake between 9 and 23 days on GGA1 was responsible for a 4.3 % decrease in feed intake. Similarly, the QTL for feed intake of dry matter between 9 and 23 days on GGA19 was responsible for a 2.7 % decrease in feed intake. When multiplied by the number of chickens per year per laying house (around 120,000), together these two QTL represent an economy of 4.91 t of feed per henhouse per year. Similarly, decreasing the dry excreta weight per bird (as for the QTL on GGA19 and 26) by 11.4 % would lead in proportion to a 5.7 % decrease in the surface needed to spread manure, since dry excreta represents 50 % of the manure [63]. The effects of these QTL on digestive efficiency can also be very important in spite of their relatively small size. For example, the positive allele of the QTL for pH in the gizzard on GGA2 leads to a 0.14 increase in the pH in the gizzard. Based on the mean value observed in our study, such a change would increase the pH to 4.0 in a region of the intestine where the activity of pepsin on plant proteins is drastically reduced [64]. Similar conclusions can be drawn using the results of the QTL for pH in the jejunum, since a shift from 6.5 to 6.3 in the pH of this region of the intestine could decrease pancreatic amylase activity by 10 % [65]. Since the experimental population used in this study originates from a commercial pure line, our results can be directly applied to this line. However, before using our results in practical selection schemes, this primo-detection experiment needs to be completed by additional data. Using a high-density SNP chip (e.g. the 60 K SNP chip) is necessary to refine the position of the QTL and to enable genomic selection on traits such as feed intake or feed efficiency. For traits, such as anatomy of the digestive tract or pH of the gizzard, which require killing birds for their measurement, using a higher density of SNPs and associating functional and positional data should help to identify genes involved in these physiological processes and to develop gene-assisted selection.

## Conclusions

Our results show that selecting birds on digestive efficiency affects a large number of genomic regions. The gene contents of these regions are consistent with the phenotypic evolutions observed between the two divergent lines, since they affect the size and structure of the gastro-intestinal tract, digestive physiology and transit time. More general processes such as energy and protein metabolism may also be involved in the differences observed between the D+ and D− lines. A transcriptomic analysis is in progress to validate or invalidate the putative candidate genes reported in this study.

## Additional files

10.1186/s12711-015-0156-y Elementary statistics of growth and excretion in F2 birds. This table presents means and standard deviations of body weight and excretion traits in the F2 population.
10.1186/s12711-015-0156-y Elementary statistics of feed intake and feed conversion ratio in F2 birds. This table presents means and standard deviations of feed intake and feed conversion in the F2 population.
10.1186/s12711-015-0156-y Elementary statistics of body composition, intestinal density, ratio of proventriculus to gizzard weights and pH of gizzard and intestine in F2 birds at 23 days. This table presents means and standard deviations of body composition, intestinal density, ratio of proventriculus to gizzard weights and pH of gizzard and intestine in the F2 population.
10.1186/s12711-015-0156-y Elementary statistics of anatomy of the digestive tract in F2. This table presents means and standard deviations of weight and length of the segments of the gut in the F2 population.

## Abbreviations

BWX: body weight at X days
FIX_Y: feed intake between X and Y days
FIDMX_Y: dry matter feed intake between X and Y days
FCRX_Y: feed conversion ratio between X and Y days
FCRDMX_Y: feed conversion ratio between X and Y days on a dry matter basis
DUW, JEW, ILW, INW: duodenum, jejunum, ileum, and small intestine weight at 23 days
DUW/BW, JEW/BW, ILW/BW, INW/BW: duodenum, jejunum, ileum, and small intestine weight at 23 days relative to body weight at 23 days
DUW/FI, JEW/FI, ILW/FI, INW/FI: duodenum, jejunum, ileum, and small intestine weight at 23 days relative to feed intake between 9 and 23 days
DUL, JEL, ILL, INL: duodenum, jejunum, ileum, and small intestine length at 23 days
DUL/BL, JEL/BL, ILL/BL, INL/BL: duodenum, jejunum, ileum, and small intestine length at 23 days relative to body length at 23 d
DUL/FI, JEL/FI, ILL/FI, INL/FI: duodenum, jejunum, ileum, and small intestine length at 23 days relative to feed intake between 9 and 23 days
DUD, JED, ILD, IND: duodenum, jejunum, ileum, and small intestine density at 23 days
FEW, DEW: fresh and dry excreta weight between 17 and 20 days
FEW/FI, DEW/FI: fresh and dry excreta weight between 17 and 20 days relative to feed intake between 17 and 20 days
PHG, PHJ, PHE: pH in the gizzard content, jejunum content and in excreta
WC: water content of excreta
