# Supplementary material for: Detection of QTL controlling feed efficiency and excretion in chickens fed a wheat-based diet
Source: Genet Sel Evol. 2015 Sep 25;47:74. doi: 10.1186/s12711-015-0156-y (PMC4582934; doi:10.1186/s12711-015-0156-y)
Supplement: Supplementary file 1 — 10.1186/s12711-015-0156-y Elementary statistics of growth and excretion in F2 birds. This table presents means and standard deviations of body weight and excretion traits in the F2 population. [file 12711_2015_156_MOESM1_ESM.docx]

**Table S1 Elementary statistics of growth and excretion in F2 birds**

| **Trait**^1^ | **Age (d)** | **N** | **Mean** | **Standard deviation** |
| --- | --- | --- | --- | --- |
| Body weight (g) | 0 | 1353 | 37.83 | 3.20 |
|  | 9 | 1047 | 135.0 | 18.7 |
|  | 14 | 933 | 175.1 | 25.4 |
|  | 17 | 930 | 244.3 | 34.3 |
|  | 20 | 931 | 334.0 | 44.9 |
| PHE | 21 | 856 | 6.476 | 0.637 |
| FEW (g) | 17-20 | 854 | 119.5 | 55.3 |
| DEW (g) | 17-20 | 856 | 46.77 | 13.01 |
| FEW/FI (g.g^-1^) | 17-20 | 849 | 76.03 | 29.34 |
| DEW/FI (g.g^-1^) | 17-20 | 846 | 33.21 | 5.13 |
| WC (%) | 17-20 | 851 | 61.02 | 11.00 |
| N/P (g.g^-1^) | 17-20 | 865 | 4.301 | 0.405 |

^1^ PHE: pH of excreta; FEW (DEW): fresh (dry) excreta weight between 17 and 20 d; FEW/FI (DEW/FI): ratio of fresh (dry) excreta weight between 17 and 20 d to feed intake between 17 and 20 d; WC: water content of excreta between 17 and 20 d; N/P: nitrogen to phosphorus ratio in excreta between 17 and 20 d
