# Supplementary material for: Detection of QTL controlling feed efficiency and excretion in chickens fed a wheat-based diet
Source: Genet Sel Evol. 2015 Sep 25;47:74. doi: 10.1186/s12711-015-0156-y (PMC4582934; doi:10.1186/s12711-015-0156-y)
Supplement: Supplementary file 2 — 10.1186/s12711-015-0156-y Elementary statistics of feed intake and feed conversion ratio in F2 birds. This table presents means and standard deviations of feed intake and feed conversion in the F2 population. [file 12711_2015_156_MOESM2_ESM.docx]

**Table S2 Elementary statistics of feed intake and feed conversion ratio in F2 birds**

|  |  | **Fresh matter basis** | | | **Dry matter basis** | | |
| --- | --- | --- | --- | --- | --- | --- | --- |
|  | **Age (d)** | **N** | **Mean** | **Std** | **N** | **Mean** | **Std** |
| Feed intake (g) | 9-14 | 929 | 116.7 | 26.8 | 929 | 100.7 | 25.3 |
|  | 14-17 | 922 | 116.3 | 23.3 | 920 | 105.1 | 21.7 |
|  | 17-20 | 927 | 158.3 | 27.7 | 921 | 143.0 | 25.9 |
|  | 20-23 | 846 | 196.5 | 32.6 | 850 | 178.4 | 31.9 |
|  | 9-23 | 811 | 586.6 | 83.9 | 819 | 526.4 | 81.9 |
|  | At slaughter^1^ | 830 | 24.75 | 5.27 |  |  |  |
|  |  | **Fresh matter basis** | | | **Dry matter basis** | | |
|  | **Age (d)** | **N** | **Mean** | **Std** | **N** | **Mean** | **Std** |
| Feed conversion ratio (g.g^-1^) | 9-14 | 907 | 3.124 | 1.098 | 891 | 2.706 | 0.984 |
|  | 14-17 | 913 | 1.745 | 0.554 | 910 | 1.527 | 0.246 |
|  | 17-20 | 924 | 1.778 | 0.234 | 918 | 1.621 | 0.346 |
|  | 20-23 | 826 | 1.688 | 0.329 | 830 | 1.545 | 0.381 |
|  | 9-23 | 811 | 1.860 | 0.206 | 819 | 1.674 | 0.208 |

^1^ Feed consumption recorded over 2h30 before slaughter, after 8 h fasting
