# Supplementary material for: Detection of QTL controlling feed efficiency and excretion in chickens fed a wheat-based diet
Source: Genet Sel Evol. 2015 Sep 25;47:74. doi: 10.1186/s12711-015-0156-y (PMC4582934; doi:10.1186/s12711-015-0156-y)
Supplement: Supplementary file 3 — 10.1186/s12711-015-0156-y Elementary statistics of body composition, intestinal density, ratio of proventriculus to gizzard weights and pH of gizzard and intestine in F2 birds at 23 days. This table presents means and standard deviations of body composition, intestinal density, ratio of proventriculus to gizzard weights and pH of gizzard and intestine in the F2 population. [file 12711_2015_156_MOESM3_ESM.docx]

**Table S3 Elementary statistics of body composition, intestinal density, ratio of proventriculus to gizzard weights and pH of gizzard and intestine in F2 birds at 23 d**

| **Trait^1^** | **N** | **Mean** | **Standard deviation** |
| --- | --- | --- | --- |
| BRY (%) | 803 | 4.172 | 1.774 |
| AFY (%) | 804 | 0.466 | 0.188 |
| PRW/GW (g.g^-1^) | 805 | 0.395 | 0.098 |
| DUD (cm.g^-1^) | 803 | 0.262 | 0.054 |
| JED (cm.g^-1^) | 803 | 0.215 | 0.034 |
| ILD (cm.g^-1^) | 803 | 0.173 | 0.032 |
| IND (cm.g^-1^) | 797 | 0.209 | 0.029 |
| PHG | 667 | 3.827 | 0.600 |
| PHJ | 798 | 6.268 | 0.189 |

^1^ BRY (AFY): breast (abdominal fat) yield; PRW/GW: proventriculus weight to gizzard weight ratio; DUD, JED, ILD, IND: density of duodenum, jejunum, ileum, and small intestine; PHG (PHJ): pH of gizzard (jejunum) content
