# Supplementary material for: Detection of QTL controlling feed efficiency and excretion in chickens fed a wheat-based diet
Source: Genet Sel Evol. 2015 Sep 25;47:74. doi: 10.1186/s12711-015-0156-y (PMC4582934; doi:10.1186/s12711-015-0156-y)
Supplement: Supplementary file 4 — 10.1186/s12711-015-0156-y Elementary statistics of anatomy of the digestive tract in F2. This table presents means and standard deviations of weight and length of the segments of the gut in the F2 population. [file 12711_2015_156_MOESM4_ESM.docx]

**Table S4 Elementary statistics of anatomy of the digestive tract in F2**

|  | **Raw organ weight (g) or length (cm)** | | | **Organ weight (g.g^-1^) or length (cm.g^-1^) relative to body weight at 23 d** | | | **Organ weight (g.g^-1^) or length (cm.g^-1^)**  **relative to feed intake between 9 and 23 d** | | |
| --- | --- | --- | --- | --- | --- | --- | --- | --- | --- |
|  | **N** | **Mean** | **Standard deviation** | **N** | **Mean** | **Standard deviation** | **N** | **Mean** | **Standard deviation** |
| Weight of |  |  |  |  |  |  |  |  |  |
| Duodenum | 803 | 6.483 | 1.525 | 800 | 1.442 | 0.317 | 773 | 1.077 | 0.248 |
| Jejunum | 804 | 10.440 | 1.816 | 801 | 2.317 | 0.322 | 774 | 1.725 | 0.252 |
| Ileum | 804 | 7.182 | 1.315 | 801 | 1.598 | 0.263 | 774 | 1.186 | 0.182 |
| Small intestine | 799 | 24.107 | 3.850 | 796 | 5.355 | 0.696 | 769 | 3.987 | 0.549 |
| Proventriculus | 806 | 3.578 | 1.043 | 803 | 0.800 | 0.240 | 776 | 0.594 | 0.185 |
| Gizzard | 805 | 9.104 | 1.536 | 802 | 2.037 | 0.369 | 775 | 1.522 | 0.327 |
| Length of |  |  |  |  |  |  |  |  |  |
| Duodenum | 803 | 0.056 | 0.009 | 803 | 0.055 | 0.009 |  |  |  |
| Jejunum | 801 | 0.109 | 0.016 | 801 | 0.109 | 0.016 |  |  |  |
| Ileum | 801 | 0.094 | 0.015 | 801 | 0.094 | 0.015 |  |  |  |
| Small intestine | 802 | 115.53 | 11.58 | 799 | 0.259 | 0.034 |  |  |  |
